# Structure and function of periplasmic nitrate reductase

**DOI:** 10.1042/BST20250033

**Published:** 2026-05-28

**Authors:** Chanakarn Mongkonpruthangkoon, Nitai C. Giri, Partha Basu

**Affiliations:** Department of Chemistry and Chemical Biology, Indiana University Indianapolis, Indianapolis, IN 46202, U.S.A

**Keywords:** DMSO reductase family, mechanism, molybdenum cofactor, nitrate reductase, operon structure, pterin conformation

## Abstract

Nitrate reductases are molybdenum cofactor (Moco)-containing enzymes that reduce nitrate to nitrite, the first step in assimilatory and dissimilatory nitrate reduction to ammonia, and in denitrification. Nitrate reductases are divided into four groups; this review will focus on periplasmic nitrate reductase (Nap), discussing the available structures of NapA in the context of other members of the DMSO reductase family. The mechanism and the roles of a specific amino acid residue are also discussed.

## Introduction and scope

Nitrate reductases are molybdenum cofactor (Moco)-containing enzymes that catalyze the reduction of nitrate to nitrite, a key reaction in the global nitrogen cycle. This process plays an essential role in denitrification and in both assimilatory and dissimilatory nitrate reduction to ammonia. While bacterial nitrate reduction occurs in the oral cavity and the gastrointestinal tract, no dedicated mammalian nitrate reductase is known [1]. However, nitrate reductions by xanthine oxidase (XO) have been reported [2,3]. Interestingly, nitrite reductions by mammalian Moco-containing enzymes have been reported, and such reactions can contribute to the NO homeostasis in mammals [4–9]. Thus, nitrate reduction is also important in the context of the microbiome and human health.

All nitrate reductases belong to the mononuclear Mo-enzyme family, in which the Mo-center serves as the catalytic core [10]. Four main types of nitrate reductases have been identified, and they differ in their subcellular localization, physiological function, and coordination sphere about the Mo center [11]. The eukaryotic nitrate reductase (Euk-NR) isolated from plants and fungi primarily assimilates nitrogen into cellular components. Three prokaryotic nitrate reductases have been identified, which include respiratory nitrate reductase (Nar), the classical respiratory nitrate reductase that couples nitrate respiration to proton translocation, cytosolic nitrate reductase (Nas), whose function is to assimilate nitrogen into the biomass, and periplasmic nitrate reductase (Nap), whose function is more complex but generally participates in dissimilatory nitrate reduction and energy dissipation [12]. Here, we examine the structure and function of Nap within the broader context of Moco-containing enzymes. Readers seeking more detailed information are encouraged to consult more comprehensive reviews [13].

## Types of nitrate reductases

**Figure 1:**
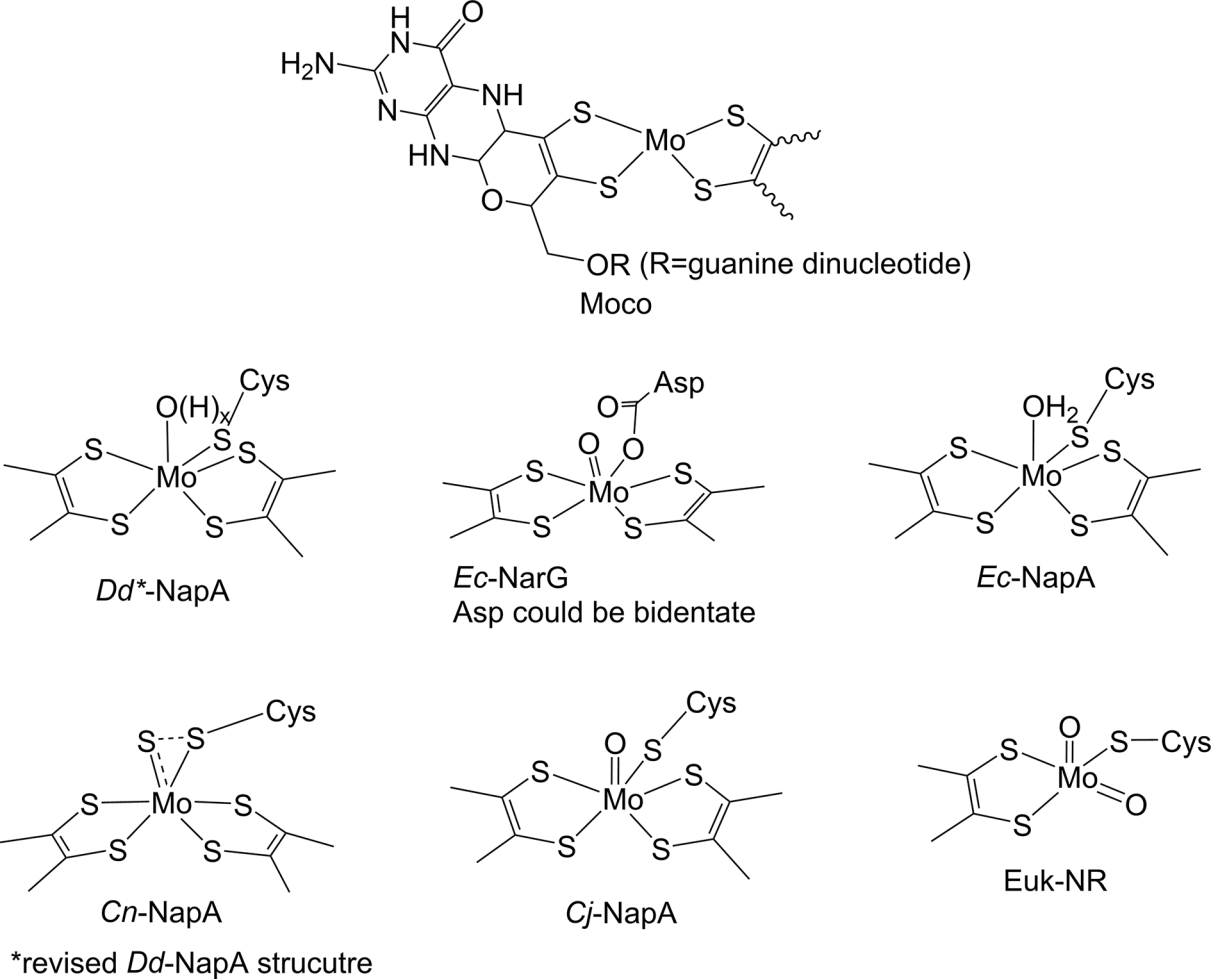
Schematic representation of the structure of Moco in Nap, and the first coordination sphere of Mo in nitrate reductases.

The Moco consists of a Mo-center coordinated by the ene-dithiolate group appended to a pterin cofactor known as pyranopterin (Figure 1) [11]. Moco-dependent enzymes are divided into three families: dimethyl sulfoxide reductase (DMSOR), XO, and sulfite oxidase (SO), exemplified by their prototype members [14]. Enzymes in the XO and SO families are found across all three domains of life, whereas DMSOR family enzymes are restricted to prokaryotes. Within this framework, Euk-NR belongs to the SO family, whereas Nar, Nap, and Nas belong to the DMSOR family.

## Overall organization

Bioinformatic analyses have identified 12 *nap* genes organized into four distinct operon structures: *napEDABC*, *napDAGHB*, *napCMADGH*, and *napAGHBFLD*, and more than one operon structure has been identified in an organism [15,16]. Among these, the gene product NapA serves as the catalytic subunit, while NapB, NapC, NapG, and NapH function as redox-active partner proteins. The gene products NapL, NapD, and NapF are involved in enzyme maturation, while NapE has been suggested to be an accessory protein and the role NapM is not clear. NapA has been isolated both as a monomer and as a heterodimer (NapAB) [17–20]. Notably, monomeric forms are generally smaller than their dimeric counterparts. The oligomeric state depends on the ability to form hydrogen-bonding interactions at the NapA:NapB interface. In *Cereibacter sphaeroides* (*Cs*), two residues in NapA (E47 and S772) have been identified as critical for heterodimer formation [21].

Phylogenetic analysis of NapA reveals that variation in primary sequence length corresponds to evolutionarily distinct clades. Smaller NapA homologs, approximately 750 amino acids in length (∼83 kDa), likely represent the ancestral form of the protein [11,22]. Increases in sequence length occurred independently at least three times during NapA divergence. Insertions of roughly 100 amino acids arose on two separate occasions: first within the Desulfurobacteriaceae family of the Aquificota phylum, and later across a broader range of NapA sequences, including those from Actinobacteriota and α-, β-, and γ-proteobacteria. Within this expanded lineage, even longer NapA variants (approximately 925–975 amino acids (aa), ∼105 kDa) evolved in groups including Campylobacterota, Thermus/Deinococcus lineages, and the Hydrogenothermaceae family of Aquificota.

## Structure

**Figure 2:**
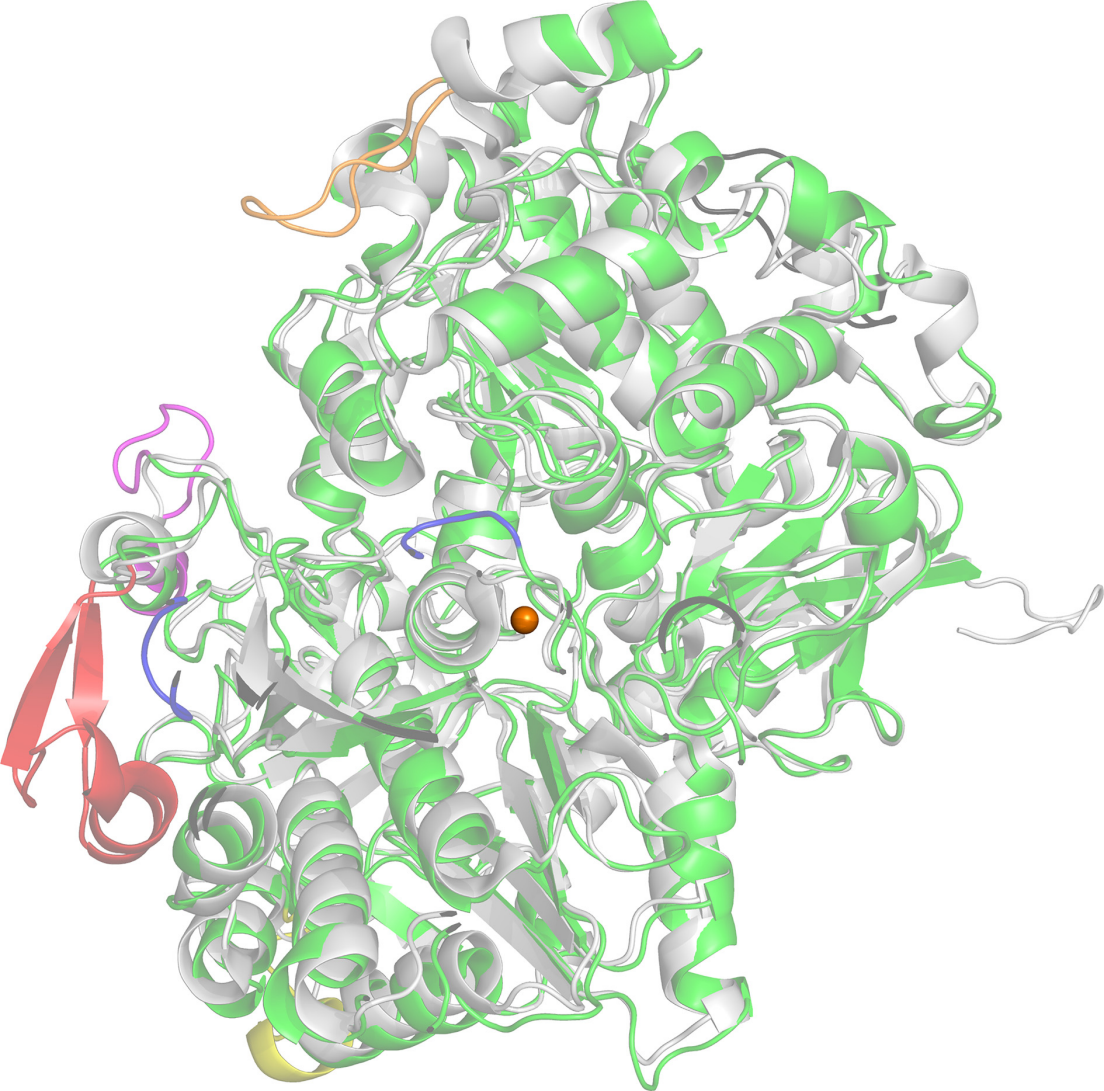
Structural alignment of *Cn*NapA (gray) with *Cj*NapA (green) shows six polypeptide inserts (blue, magenta, orange, red, yellow, and pale cyan) in *Cj*NapA. Mo is shown as a sphere.

NapA from five different organisms has been structurally characterized. These five NapAs can be divided into three groups based on the number of aa and hence the molecular weight: *Desulfovibrio desulfuricans* (*Dd*) NapA is the smallest (755 aa), *Escherichia coli* (*Ec*) NapA (828 aa), *Cs*NapA (831 aa), *Cupriavidus necator* (*Cn*) NapA (831 aa) are in the medium size category, and *Campylobacter jejuni* (*Cj*) NapA (924 aa) is the largest. Of these, *E. coli*, *C. necator*, and *C. spharoides* NapAs were isolated as heterodimers. While *Ec*NapAB was isolated as a heterodimer, the crystal structure of the monomeric NapA was determined. The overall structures of monomeric and heterodimeric NapAs are similar (Figure 2) [18,22,23]. These enzymes contain an N-terminal 4Fe4S cluster as a partner prosthetic group. The Mo center is deeply buried inside the protein, with a substrate channel of ∼15 Å, which can influence its properties [24–26]. In dimeric enzymes, the substrate channel is oriented opposite to the heterodimer contact interface.

In all five structures, the Mo center is ligated by four thiolates from two pterin rings and one cysteine from NapA (Figure 1) [17–19,22,23]. However, there are some differences in the assignment of the terminal sixth ligand. In the first structure from *Dd*NapA (1.9Å resolution), the sixth ligand was assigned to be water or hydroxo (Mo-O distance of 2.1Å) (Figure 1) [18]. This water-based ligand is pointed toward the substrate channel, which contains a few charged amino acid residues (Arg354, Asp155, Glu156, and Asp355). Arg354 is almost above the catalytic pocket and may provide a place for nitrate binding.

In later structures, the terminal oxo group was absent; instead, it was replaced by an S-based ligand, leading to the so-called ‘sulfur shift’ mechanism [27]. In *Cn*NapAB, both oxidized and partially reduced states show a Mo = S moiety with minimal perturbation [20]. The partially reduced form exhibited two alternative conformations of the Mo-coordinating cysteine. However, the low occupancy of one conformation (12% vs 88%) suggests an unbound cysteine. Both the (Mo)S—S(cys) and Mo = S distances (2.7Å and 2.4Å, respectively) are longer than typical distances found in small molecules (from CCDC, mean d(S—S) = 2.05 ± 0.06 Å, and mean d(Mo = S) = 2.15 ± 0.04 Å). However, a disulfide bond between a cysteine sulfur and a terminal sulfido group can be formed via internal redox [28].

In the *Ec*NapA, the sixth ligand is assigned as water, which is consistent with Mo-O distance of 2.6 Å. The longer Mo-O bond may allow H-bonding interaction with Gln374. Thus, this residue might play a role in nitrate reduction. Also, a water molecule is located close to the Mo-center that may be involved in H-bonding interactions with the 4Fe4S cluster and the pterin ring. A similarly located water molecule is also present in *Dd*NapA and formate dehydrogenase (Fdh) [29]. Thus, this water-mediated H-bonding interaction might facilitate electron transfer between the 4Fe4S cluster and Moco.

The surface of *Ec*NapA in the NapB binding region is less polar and anionic than that of α-proteobacteria, such as *C. sphaeroides*. This may explain the weaker NapA:NapB interaction in *E. coli* compared to *Cs*NapA:NapB. Also, the surface that surrounds the 4Fe4S cluster in *Ec*NapA is less anionic. More negative surface potential makes the reduction of the 4Fe4S cluster more difficult [17]. This is consistent with a more negative redox potential (−250 mV) in *Cs*NapA compared to −25 mV in *Ec*NapA for the FeS^2+/1+^ couple [17,19].

The catalytic subunit of the *Cs*NapAB structure is similar to that of *Dd*NapA. The conformation of pterin rings is also similar. The structure shows extensive interactions between the two subunits, which can explain the high affinity observed in kinetic measurements. However, the terminal ligand could not be identified due to insufficient structural resolution [19]. The *Ec*NapAB exists as a weak heterodimer with dissociation constants (K_d_) 15 and 32 μM for oxidized and reduced complexes, respectively, while *Cs*NapAB has a much stronger affinity (K_d_ = 0.5 nM) [17,19]. The stability of the dimer is due to hydrogen bond/salt bridges at the NapA:NapB interface, as seen in the *Cs*NapAB, where residues E47 and S772 in NapA are critical for dimerization [21].

In the NapAB heterodimer, the prosthetic groups form an electron-transport chain from the presumed electron-entry point at heme I to the Mo-center. The redox cofactors align so that distances between any two cofactors are less than 14 Å, ensuring efficient electron transfer. In *Cn*NapAB and *Cs*NapAB, the two hemes of NapB are parallel to one another, and the two iron atoms are 10 Å and 9.7 Å apart, respectively, and the porphyrin rings are within the van der Waals contact [19,23]. The NapB's proximal iron center (heme II) is ∼7.5 Å from the NapA tyrosine (Tyr 58), which is conserved in all heterodimeric dimeric nap. Therefore, it has been suggested to play a role in intersubunit electron transfer. However, experimental evidence is lacking.

We recently reported CryoEM structure of *Cj*NapA [22]. The overall structure of *Cj*NapA is similar to that of other structurally characterized NapA, with an additional ∼100 amino acid residues. These additional amino acid residues constitute several polypeptide insertions. Three of these positively charged inserts are part of the substrate channel. These positively charged inserts can serve as docking sites for nitrate, resulting in its high affinity. Although the CryoEM structure cannot resolve the identity of the terminal ligand, X-ray absorption spectroscopy (XAS) experiments done on fully oxidized *Cj*NapA indicate the presence of an oxo group as the sixth ligand [30]. The absence of a terminal sulfido ligand has also been supported by biochemical experiments [31].

**Figure 3:**
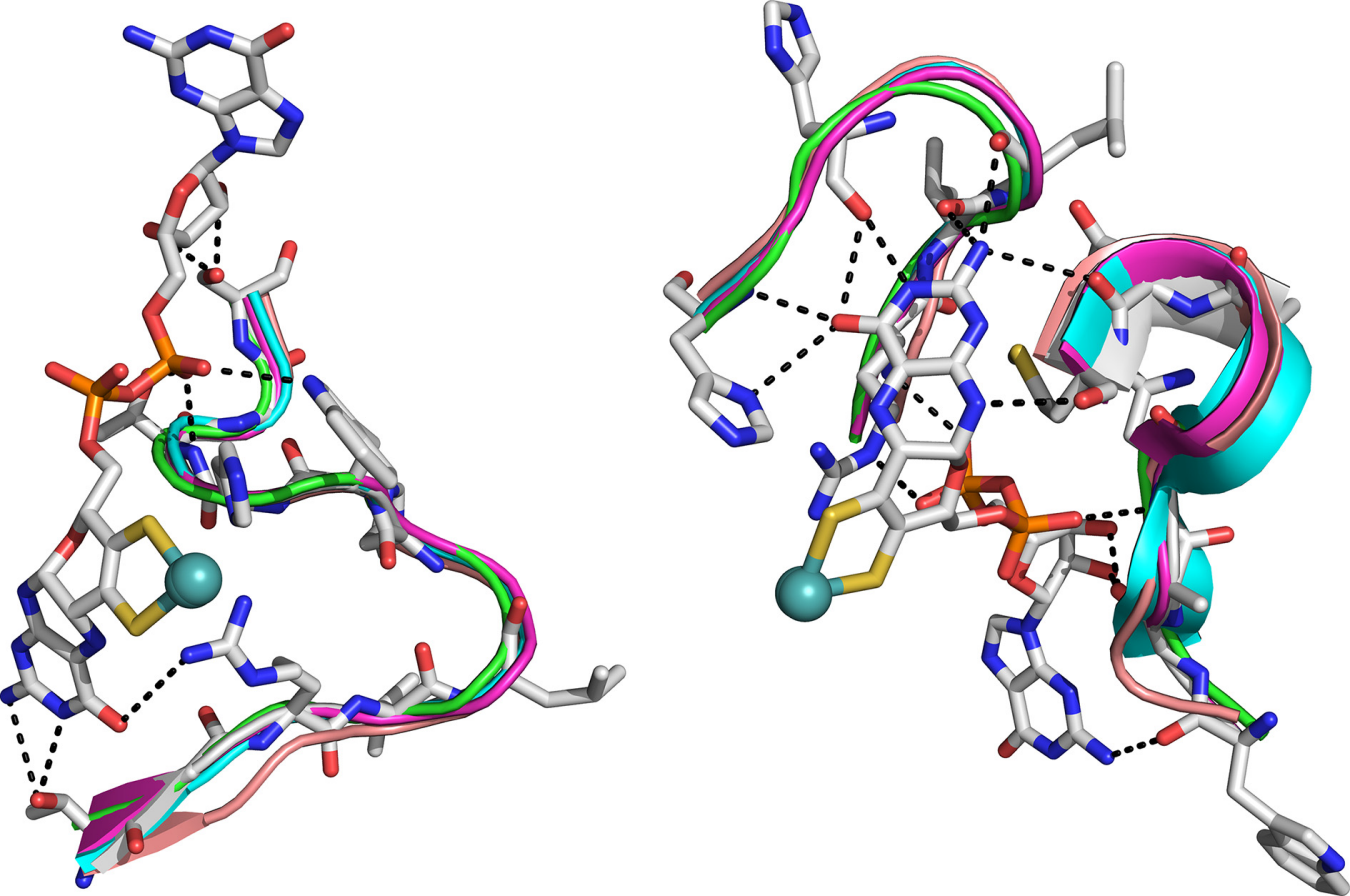
Interactions of amino acid residues with proximal pterin (left) and distal pterin (right). For clarity, only the pterin rings and their interactions with the amino acid residues of EcNapA are shown. However, the cartoon of the patches of amino acids in all five NapAs (shown in different colors) is very similar.

The pterin moiety of Moco is stabilized by extensive H-bonding with the protein in addition to π-stacking interaction. Although amino acids from different domains are involved in these interactions, there are two patches of amino acids that form numerous H-bonds with the pterin units: proximal and distal refer to closer or farther from the 4Fe4S cluster. Both patches interact with the proximal pterin while only one interacts with the distal pterin. One of these patches includes amino acid residues 212 to 219 (*Ec*NapA UniProt numbering) (Figure 3). Among these residues, Gly213, Asn215, and Glu218 are conserved, while Trp212, Met180, and Met183 are present in all structurally characterized NapAs except *Dd*NapA. The second patch includes residues 718 to 729 (*Ec*NapA UniProt numbering). Among these residues, Arg720, Val721, His724, Trp725, and His726 are conserved, while Thr718 is present in all four NapAs except *Dd*NapA, where it is replaced with a Ser. Similarly, Leu722 is present in all four NapAs except *Dd*NapA where an Ile takes its place. The Thr727 and Ser729 can be either a Thr or a Ser.

Although monomeric and heterodimeric NapAs share a similar overall structure, the identity of the sixth ligand has been debated. This question extends beyond NapA to other enzymes in the DMSOR family. For example, *E. coli* trimethylamine N-oxide reductase (Tor) has been proposed to contain a terminal sulfido ligand in anaerobically grown cells [32], and structural studies of *E. coli* Fdh support a terminal Mo = S rather than the originally assigned Mo-OH moiety [33]. However, while the Fdh operon includes a sulfur transferase gene, such a gene is absent from the *nap* and *tor* operons, implying that any sulfido incorporation would require a protein encoded elsewhere.

Experimental evidence further informs this debate. In XO, a terminal sulfido ligand is confirmed by cyanide treatment, which produces thiocyanate (^−^SCN) and irreversibly inhibits the enzyme [34]. In contrast, biotin sulfoxide reductase (BSOR) shows reversible cyanide inhibition, consistent with the absence of a cyanolyzable sulfur [35], a conclusion supported by XAS and resonance Raman data indicating an oxo ligand [36,37]. Similarly, biochemical studies of *Cj*NapA detect no cyanolyzable sulfido group [31]. Its noncompetitive, non-suicidal inhibition by cyanide and supporting XAS data both point to an oxo, rather than sulfido, ligand at the Mo center. Taken together, the available evidence supports assignment of an oxo group as the sixth ligand in NapA.

## Role of a conserved lysine

**Figure 4:**
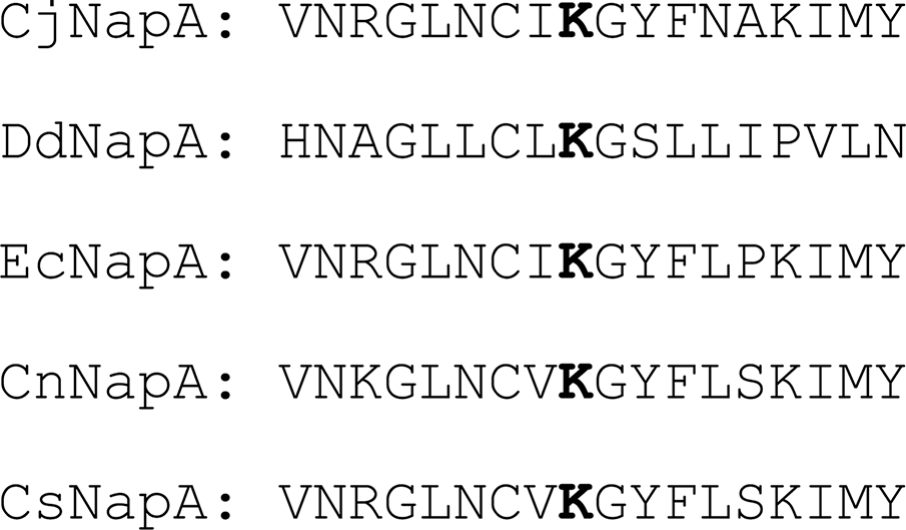
Conserved Lys (in bold) in NapAs from different organisms.

The structures of NapAs show that a Lys residue lies at the midpoint of the edge of the Moco, and the 4Fe4S cluster, and connects these two prosthetic groups via H-bonding interactions. This Lys is conserved in several members of the DMSOR family of enzymes, including Fdh, ethylbenzene dehydrogenase (Ebdh), and perchlorate reductase (Pcr) [38–40]. In these structures, a water molecule was located between the Lys residue, the 4Fe4S cluster, and the proximal pterin. A water molecule is conserved in all available structures of NapA (except *Cs*NapA, where no water molecule was described), as well as closely related Fdh (Figure 4). Substitution of conserved Lys79 in *Cj*NapA with Ala results in complete loss of activity, confirming its role in catalysis [41]. NapA is sensitive to inhibition by various anions, such as cyanide, thiocyanate, perchlorate, and azide [42,43]. Detailed studies with *Cj*NapA show that perchlorate inhibits competitively, cyanide non-competitively, and thiocyanate and azide uncompetitively [41]. Thus, the inhibition of NapA by these anions, except perchlorate, does not involve direct binding to the Mo center. It was suggested that the inhibition of NapA is caused by the replacement of conserved water by these anions,. This water molecule connects Lys79, the Moco, and the 4Fe4S cluster via an H-bonding network. It is possible that the reason for the loss of activity of the K79A variant and the inhibition of NapA lies in the disruption of this H-bonding network and the consequent impairment of electron transfer and/or proton transfer.

## The conformation of the pterin

In NapA, two tricyclic pyranopterin ene-dithiolate moieties coordinate the Mo- center and are designated as proximal or distal. Bicyclic pterins have also been identified in enzymes, such as Ebdh, nitrite oxidoreductase (NxrA), PcrA, and NarG, although the functional significance of bicyclic versus tricyclic forms remains unclear. Previous analyses of over 100 mononuclear molybdenum and tungsten enzyme structures have shown that the pyranopterin conformation can be described using two dihedral angles, α and β [44]. Plots of β versus α provide insight into the degree of planarity or distortion of the pterin. These studies indicate that the two pterins in DMSOR family enzymes are conformationally distinct. The proximal pterin consistently exhibits greater distortion than the distal pterin.

The distortions of the pterin rings have been correlated with their redox states (e.g. dihydro or tetrahydro forms) [44]. The dihedral angles of the distal pterin are like those of dihydropterins, suggesting that the former may adopt a dihydro form. In contrast, the proximal pterin exhibits a broader range of dihedral values. For example, the proximal pterin in *Cj*NapA more closely resembles that of *Cs*NapA, whereas those in *Cn*NapA, *Ec*NapA, and *Dd*NapA are more like one another. However, directly correlating pyranopterin redox states with crystallographic structures remains challenging. Pushie et al. [45], noted limitations in structural resolution, as well as the influence of the metal center and the surrounding protein environment, including hydrogen-bonding interactions, can contribute to observed distortions. Consequently, caution is warranted when drawing definitive conclusions.

The proximal pterin is generally considered to participate in electron transfer, whereas the distal pterin is thought to modulate the redox potential of the Mo-center. For instance, the dihedral angles of the proximal pterin in *Cj*NapA are close to those calculated for tetrahydropterin, supporting a potential role in electron transfer to the Mo center. Additional support for this assignment comes from a conserved Lys residue located between the proximal pterin and the 4Fe4S cluster. Substitution of this Lys with alanine or methionine results in a loss of nitrate reductase activity, further underscoring its functional importance. However, the distinct clustering of the distal pterin and the more dispersed distribution of the proximal pterin in the α/β plot suggest that the proximal pterin may also contribute to redox tuning. This interpretation is consistent with the wide variation in Mo(VI/V) redox potentials observed among NapAs, ranging from −100 to 100 mV in *Ec*NapA to values exceeding 300 mV in *Cs*NapA [17,19].

In DMSOR family members, the proximal pterin is in the reduced tetrahydro form, and the distal one is in oxidized dihydro form. However, BamB, a W-dependent benzoyl CoA reductase, contains both pterin rings in reduced tetrahydro form [46]. To understand the influence of pyranopterin oxidation state on BamB-catalyzed reaction, QM/MM calculations were performed. The results indicate that the energy barrier is 4 kcal/mol higher when both pyranopterins are oxidized. When one pterin is oxidized, and the other one is reduced, the energy barrier is lower than when both pterins are reduced. However, under this condition (one pterin oxidized and one pterin reduced), the enzymatic reaction becomes irreversible, which is inconsistent with the experimental observation [47]. Thus, two reduced pyranoptein is essential for the reversibility of the enzyme reaction [48]. Similar investigations on other DMSOR family enzymes are necessary to determine whether this is an exception or a trend.

## Mechanism

The nitrate reductases are considered to be an oxotransferase, meaning in the reduction of nitrate to nitrite and the oxidation of the Mo(IV) center to oxo-Mo(VI) follows an oxygen atom transfer (OAT) process. This reaction was demonstrated using an ^18^O-labeling experiment with *Cj*NapA [31]. Use of N^18^O_3_^−^ as a substrate generates a Mo^18^O center, and the labeled oxygen is transferred to a tertiary phosphine, producing the phosphine oxide, which was detected by mass spectrometry. The kinetic isotope effect is consistent with the oxygen atom being the rate-limiting step. The oxidized NapA was also found to oxidize nitrite to nitrate. The ^18^O labeling experiment demonstrated that this oxygen originated from water. However, this reverse reaction is less efficient than the forward reaction.

**Figure 5:**
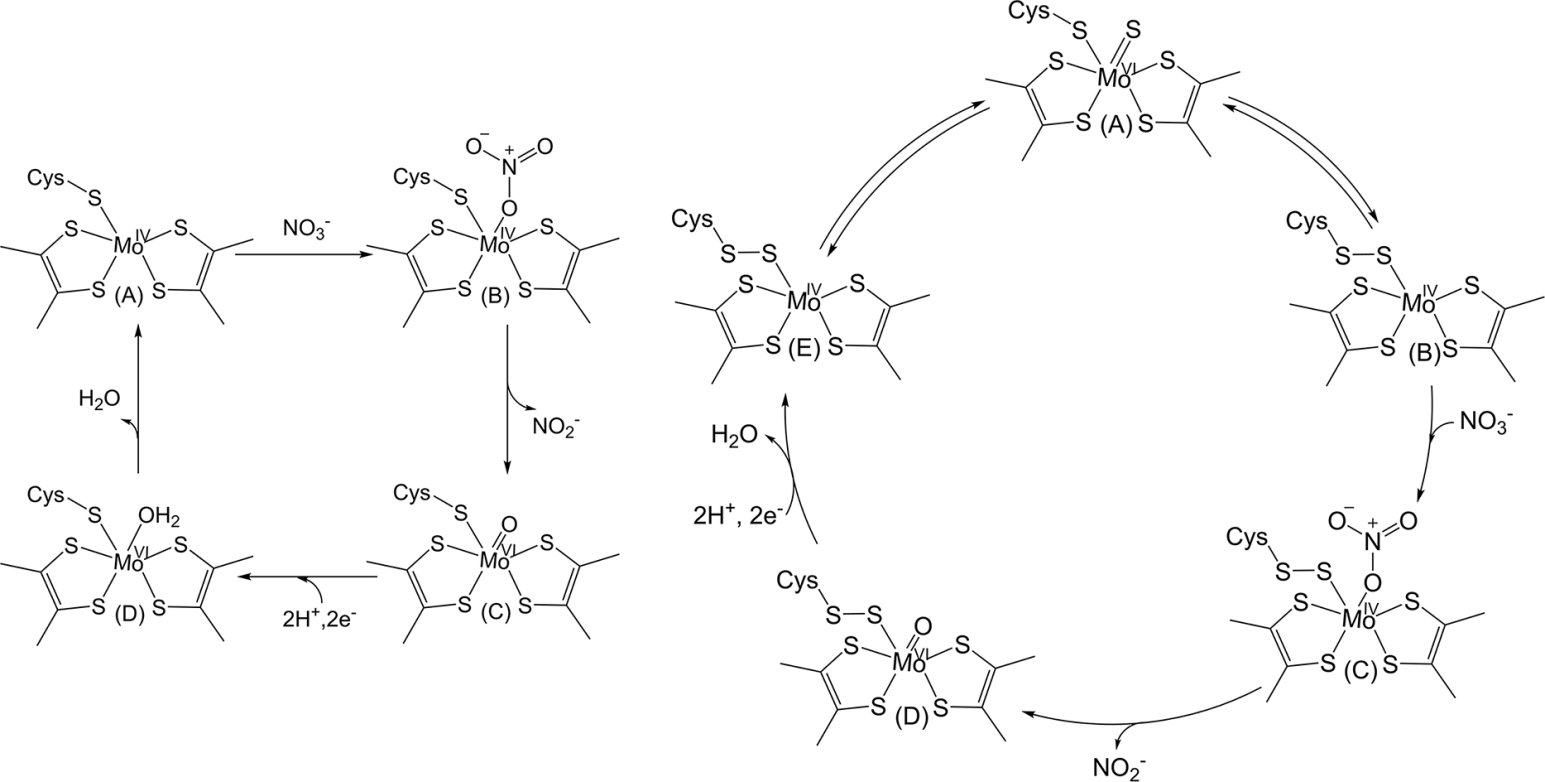
Left: the initial mechanism proposed for *Dd*NapA; right: revised sulfur-shift mechanism.

Over the years, several structure-based mechanisms have been proposed for NapA. In all cases, the mechanism involves nitrate reduction at the Mo center where nitrate transfers one of its oxygen atoms. Based on the *Dd*NapA structure, a mechanism involving OAT was proposed for nitrate reduction (Figure 5) [18]. This mechanism is consistent with the OAT reaction proposed for DMSOR and arsenate reductase (ArrAB) [49,50]. A water-coordinated Mo(VI) center has been suggested as a stable species, even though such a water molecule is likely to have a low pK_a_, leading to dissociation of a proton under physiological pH [51].

The ‘sulfur-shift’ mechanism (Figure 5) revises this mechanism, in which a terminal sulfido group forms a persulfide bond with cysteine sulfur that has dissociated from the Mo center [52]. In this mechanism, Mo(VI) centers gain an oxo group from the substrate. This is also inspired by the crystal structure showing a terminal sulfido group rather than a terminal oxo-group at the oxidized Mo center. An updated S-shift mechanism proposes that the initial state of the enzyme has an Mo—S^−^ as opposed to the Mo = S moiety, and no state has a discrete Mo = S bond [27]. This mechanism was supported by density functional theory calculations [53,54]. It is not clear how the disulfide bond remains intact during the reductive regeneration steps.

**Figure 6:**
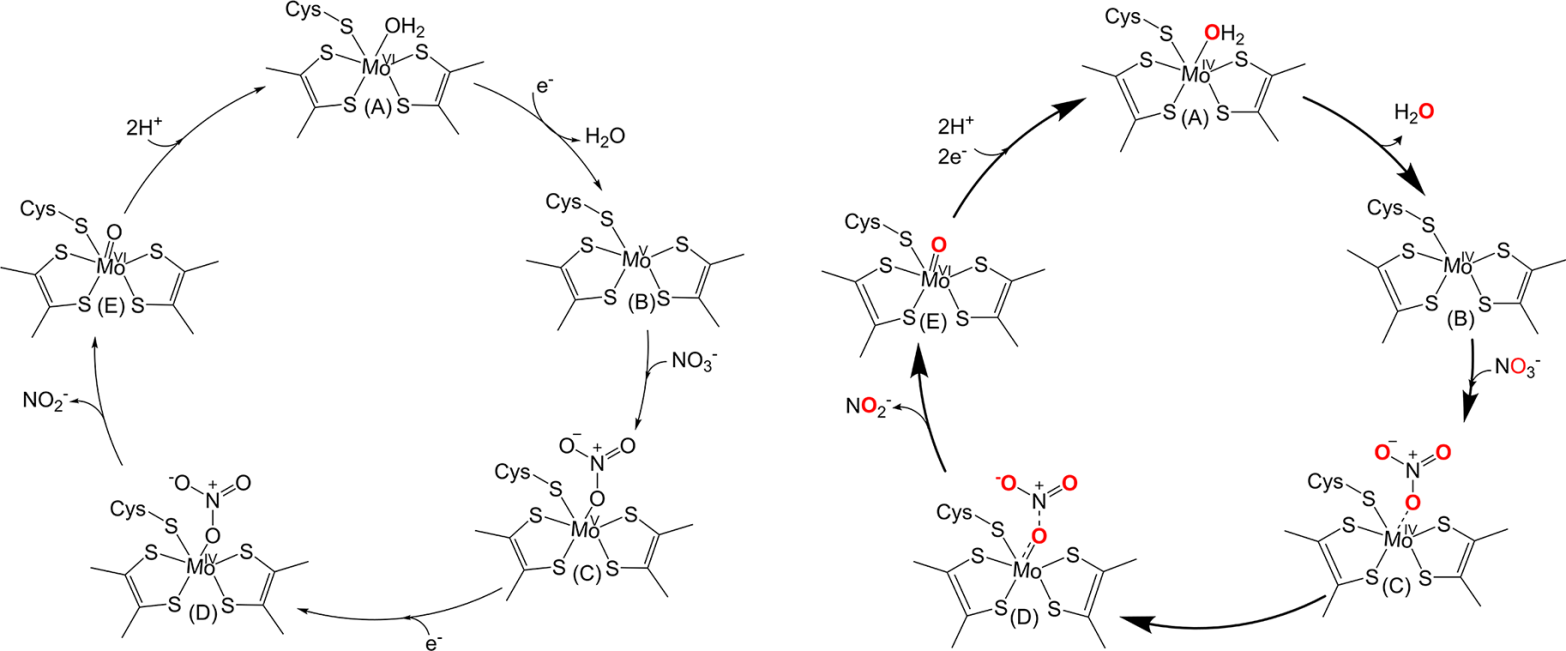
The proposed mechanism for EcNapA (left) and CjNapA (right).

The third mechanism (Figure 6) for *Ec*NapA involves nitrate binding to Mo(V), followed by reduction to Mo(IV) and OAT, forming an oxo-Mo(VI) center and nitrite [17]. Like the first mechanism, it includes a water molecule ligated to Mo(VI). However, as the authors note, there is uncertainty regarding the oxidation state of the Mo center. Thus, it suggests that the Mo(IV) form cannot bind the substrate.

The *R. capsulatus* Fdh, FdsA, contains a terminal sulfido group [55]. While the sulfido form is needed for formate oxidation, both sulfido and oxo forms can reduce nitrate, with the oxo form showing a higher turnover number for nitrate reduction, suggesting the sulfido ligand is not essential for nitrate reduction. The biochemical and spectroscopic evidence indicate that *Cj*NapA lacks a terminal sulfido ligand, which is inconsistent with the proposed S-shift mechanism. As-isolated NapA contains the Mo ion in higher oxidation states, i.e. Mo(V) or Mo(VI) [17,18,56,57]. However, reduction of isolated *Ec*NapA with dithionite or reduced methyl viologen (MV^r^) led to the disappearance of the Mo(V) signal [58]. The fully reduced enzyme lacks any terminal oxo group and has a desoxo-Mo(IV) species [58]. Single-turnover experiments with the as-isolated *Cj*NapA showed no nitrite formation upon incubation with excess nitrate. However, fully reduced *Cj*NapA under similar conditions produced nitrite. Because no reducing equivalents were provided after nitrate was added to the fully reduced enzyme, this result is inconsistent with the mechanism proposed for *Ec*NapA (Figure 6). Interestingly, the computational modeling of Mo(V) coordinated with NO_3_^−^ does not indicate any activation of the nitrate by the single electron at the Mo(V) site. These results imply that reduction to the Mo(IV) state is required for nitrate reduction and support the mechanism proposed for *Dd*NapA (Figure 5, left), with some modifications, as shown for the *Cj*NapA mechanism (Figure 6, right) [31].

## Summary

Significant progress has been made since the initial report of the crystal structure of periplasmic nitrate reductase from *D. desulfuricans* by Dias et al. [18] Subsequent studies have greatly advanced our understanding of Nap systems, including the diversity of *nap* operon architectures, the structural variability among NapA enzymes, and their catalytic properties, reactivity, and inhibition profiles. In addition, insights into cofactor coordination, electron transfer pathways, and substrate interactions have begun to clarify the mechanistic principles underlying Nap function. Despite these advances, important questions remain. Ongoing structural, spectroscopic, and computational studies are expected to uncover new NapA variants and further refine our understanding of their catalytic mechanisms. At the same time, the physiological roles of NapA across organisms, particularly under varying environmental conditions, are likely to be clarified.

## Perspectives

- Biological nitrate reduction is a key reaction in the biogeochemical cycle and has important implications for human health. Studying the reaction and the enzymes involved can elucidate the mechanisms that drive the nitrogen cycle and the survival of pathogenic bacteria.
- Since the first crystal structure of Nap was resolved, studies have advanced our understanding of Nap systems, including the diversity of *nap* operon architectures, structural variability among NapAs, and the catalytic properties, reactivity, and inhibition profiles of NapA. Moreover, insights into cofactor coordination, electron pathways, and substrate interaction are unveiling how Nap functions.
- Ongoing structural, spectroscopic, and computational studies will likely uncover new NapA variants and refine our understanding of their catalytic mechanisms and physiological roles across organisms. Additionally, the versatility of Nap enzymes to catalyze OAT reactions makes them an ideal candidate for future biotechnological applications, including bioremediation and green catalytic processes.

## Open Access

Open access for this article is provided via our Subscribe to Open programme, made possible through the support of our subscribing institutions. Learn more here: https://portlandpress.com/pages/open_access_options_and_prices#conditional.

## Abbreviations

BSOR: Biotin sulfoxide reductase
DMSOR: Dimethyl sulfoxide reductase
Ebdh: Ethylbenzene dehydrogenase
Fdh: Formate dehydrogenase
Moco: Molybdenum cofactor
OAT: Oxygen atom transfer
Pcr: Perchlorate reductase
SO: Sulfite oxidase
Tor: Trinethylamine N-oxide reductase
XAS: X-ray absorption spectroscopy
XO: Xanthine oxidase
